# Sparse Reconstruction for Bioluminescence Tomography Based on the Semigreedy Method

**DOI:** 10.1155/2012/494808

**Published:** 2012-08-14

**Authors:** Wei Guo, Kebin Jia, Qian Zhang, Xueyan Liu, Jinchao Feng, Chenghu Qin, Xibo Ma, Xin Yang, Jie Tian

**Affiliations:** ^1^The College of Electronic Information & Control Engineering, Beijing University of Technology, Beijing 100124, China; ^2^Intelligent Medical Research Center, Institute of Automation, Chinese Academy of Sciences, Beijing 100190, China; ^3^Sino-Dutch Biomedical and Information Engineering School of Northeastern University, Liaoning, Shenyang 110004, China

## Abstract

Bioluminescence tomography (BLT) is a molecular imaging modality which can three-dimensionally resolve the molecular processes in small animals *in vivo*. The ill-posedness nature of BLT problem makes its reconstruction bears nonunique solution and is sensitive to noise. In this paper, we proposed a sparse BLT reconstruction algorithm based on semigreedy method. To reduce the ill-posedness and computational cost, the optimal permissible source region was automatically chosen by using an iterative search tree. The proposed method obtained fast and stable source reconstruction from the whole body and imposed constraint without using a regularization penalty term. Numerical simulations on a mouse atlas, and *in vivo* mouse experiments were conducted to validate the effectiveness and potential of the method.

## 1. Introduction

Due to its ability of monitoring physiological and pathological activities at the molecular level, small animal optical molecular imaging has become an important method for biomedical research. Bioluminescence imaging, as one of optical molecular imaging modalities, has attracted attention for its advantages in sensitivity, specificity, and cost effectiveness in cancer research and drug development [1–3]. Compared with planar bioluminescent imaging, BLT can three-dimensionally reconstruct the bioluminescent probes in small animals [4].

The generic BLT model is ill-posed. However, it has been theoretically proven that the solution uniqueness can be established under practical constraints using a *priori* knowledge [5]. In most existing reconstructions, multi spectral measurement [6–10], permissible source region (PSR) [9–12], and sparse reconstruction [13–15] are three common strategies to reduce ill posedness of BLT. Although multi spectral techniques improve reconstruction qualities to a certain degree by increasing the measurable data, they in turn impose some limitations in practical applications such as increased signal acquisition time and a high computational cost [16]. Besides, the PSR strategy can significantly improve the location accuracy of reconstructed source and reduce the computational cost by limiting the reconstruction region into a small area. However, in practical applications, both the size and position of the permissible region have significant impact on imaging results [5]. Additionally, since the bioluminescent source distribution is usually sparse in practical applications and only insufficient boundary measurements are available, the compressed sensing can bring benefits in spatial resolution and algorithm stability to BLT reconstruction. Recently, many sparse reconstruction methods have emerged in BLT [17–19]. The majority of them reformulate the BLT inverse problem into minimizing an objective functions that integrate a sparse regularization term with a quadratic error term and solve it via computationally tractable convex optimization methods, such as linear programming and gradient methods, However, the efficacy of the sparse regularization methods strongly depends on the choice of regularization parameter in practical applications [19].

The existing works have demonstrated that PSR can improve reconstruction qualities by reducing the number of unknown variables. Although the meaningful results can be obtained by using the PSR that is based on the bioluminescent signals and *a priori* knowledge available from a specific biomedical application [4, 11, 12], in most cases, it is rather difficultly to manually select such a small and appropriate region. Recently, some optimal permissible source region methods have emerged in BLT. Feng et al. presented a reconstruction algorithm for a spectrally resolved BLT based on an adaptive rough estimate of an optimal permissible source region and multilevel finite element method approach (FEM), where Tikhonov regularization was used to solve the constrained BLT inverse problem [9]. Naser and Patterson proposed a two-step reconstruction algorithm of bioluminescence, in which the permissible regions were shrunk by using an iterative minimization solution based on the L1 norm [10]. These works demonstrated the feasibility and potential of the optimal PSR techniques with numerical simulation. However, both of two previous reconstructions also needed the regularization methods to reconstruct the sources in the allowed region, which made the reconstruction results also depend on the choice of regularization parameter. Furthermore, they were demonstrated with only regular phantoms simulations and presented no *in vivo* experiment validation.

In this work, a novel BLT reconstruction algorithm based on the semi greedy method was proposed. The optimal PSR problem was cast into a search for the correct support of source distribution among a number of dynamically evolving candidate subsets, and the optimal PSR was chosen automatically by using an iterative search tree. Therefore, the columns of the system matrix were treated as the nodes for building up the search tree where each path from the root to a leaf node denoted a candidate. The search tree was initialized with some unspecific nodes. At each iteration, new nodes were appended to the most promising path, which were selected to minimize the cost function based on the residue. The permissible source region was expended by adding nodes with high a probability to contribute to the source. Among the system matrix, the columns that were corresponding to the nodes contained in the most promising path were selected to obtain the source distribution. By automatically choosing an optimal PSR, the method reduced the ill posedness of the problem and imposed constraint without using a regularization penalty term.

This paper is organized as follows. In Section 2, the forward photon propagation model, the inverse problem for BLT with FEM, and the proposed algorithm are introduced. In Section 3, the numerical simulations in a mouse atlas demonstrate the performance of the proposed method. In Section 4, an *in vivo* mouse experiment is conducted to further evaluate its reliability. Finally, we discuss the results and conclude this paper.

## 2. Method

Light propagation in biological media is essentially the transport of radiant energy. The radiative transfer equation (RTE) can rigorously describe light transport in turbid media [20]. Compared with the other approximations of RTE such as simple spherical harmonics, spherical harmonics and discrete ordinates, the following steady-state diffusion approximate equation (DA) in (1) is the most popular one as a result of its moderate computational efficiency and explicit physical meaning [4, 5, 11, 15, 21]. Assuming that the bioluminescence imaging experiment is performed in a totally dark environment and no photon travels into *Ω* through the boundary ∂*Ω*, the equation is subject to the Robin boundary condition in (2) as follows:

(1) $$\begin{array}{c} -\nabla \cdot D\left(r\right)\nabla \Phi \left(r\right)+\mu_{a}\left(r\right)\Phi \left(r\right)=S\left(r\right)\;\left(r\in \Omega \right), \end{array}$$

(2) $$\begin{array}{c} \Phi \left(r\right)+2A\left(r;n,n^{\prime }\right)D\left(r\right)\left(v\left(r\right)\cdot \nabla \Phi \left(r\right)\right)=0\;\left(r\in \partial \Omega \right), \end{array}$$

where *Ω* is the domain of the problem, *S*(*r*) donates the source energy distribution, Φ(*r*) represents the photo fluence rate, *μ*_*a*_(*r*) is the absorption coefficient, *μ*_s_′(*r*) is the reduced scattering coefficient, *D*(*r*) = 1/3(*μ*_*a*_(*r*) + *μ*_s_′(*r*)) indicates the optical diffusion coefficient, ∂*Ω* donates the boundary of the problem, and *A*(*r*, *n*, *n*′) represents the mismatch coefficient between *Ω* and its surrounding medium. The measured quantity on the boundary ∂*Ω* is given by the outgoing radiation as follows:

(3) $$\begin{array}{c} Q\left(r\right)=-D\left(r\right)\left(v\left(r\right)\cdot \nabla \Phi \left(r\right)\right)=\frac{\Phi \left(r\right)}{\left(2A\left(r;n,n\right)\right)}\;\left(r\in \partial \Omega \right). \end{array}$$

FEM is a powerful tool for solving the DA equation [4, 6–10]. By using FEM to discretely approach the solving domain and making a series of transformations and rearrangements, the linear relationship links the source distribution inside the heterogeneous medium, and the photon fluence rate on the surface is established as follows:

(4) $$\begin{array}{c} M\Phi =FX, \end{array}$$

where *X* is the source distribution of the interior nodes, Φ is the measurable photon flux photon on the boundary nodes, *M* is the positive definite matrix, and *F* is the source weight matrix. The nonmeasurable entries in Φ and corresponding rows in *M* ^−1^ *F* can be removed. Then a new linear relationship can be obtained as follows [4, 22]:

(5) $$\begin{array}{c} AX=\Phi^{m}. \end{array}$$

For BLT, the domains of the bioluminescent sources are usually very small and sparse compared with the entire reconstruction domain. That means that there are only fewer nonzero components in *X*. Therefore, the system matrix *A* can be seen as the dictionary, and Φ^*m*^ has a less-term representation over the dictionary. As a consequence, the aim of the proposed method is imposing the constraint on the source space by choosing only the part that contributes to the source distribution. In the language of sparse approximation, greed pursuit algorithms are useful methods for solving this problem [23, 24]. For instance, Orthogonal Matching Pursuit (OMP) is to pick columns in a greedy fashion [25]. According to the introduction of OMP, the reconstruction starts with an empty index set. At each iteration, we choose the single column of *A* that is most strongly correlated with the remaining part of Φ^*m*^. Then, we subtract its contribution to Φ^*m*^ and iterate on the residual. The reconstruction is stopped after a number of iterations. Unfortunately, from experimental results shown in Figure 2 and Table 2, we found that OMP, as the single-path algorithm, could not achieve the desired expectations for reconstructing the bioluminescent source. In the experiments, when the computation of a single path selects a wrong column, the correct one is still in the set of candidate representations. Therefore, incorporation of a multipath search strategy is motivated to improve reconstruction. In this section, the semigreedy method was used to search for the correct support of Φ^*m*^ among a number of dynamically evolving candidate subsets.

A general best first (GBF) is a search algorithm which constructs a tree *T* by expanding the most promising node chosen according to a specified rule. Search algorithm A*, as one of the most studied versions of GBF, can find path in combinatorial search. It selects an optimal path by minimizing an additive evaluation function *f*(*n*) = *g*(*n*) + *h*(*n*), where *g*(*n*) is the cost of the currently evaluated path from start node *s* to *n*, and *h*(*n*) is a heuristic estimate of the cost of the path remaining between *n* and some goal nodes [26–29]. In our problem, the A* search tree was iteratively built up by nodes which represent the dictionary atoms. Each path from the root to a leaf node denoted a subset of dictionary atoms which was a candidate support for Φ^*m*^. Let us define the notation. *S*_*i*_ and *C*_*i*_ denote the atoms contained in path *i* and the vector of corresponding coefficients obtained after orthogonal projection of the residue onto the set of selected columns [25]. Similarly, *s*__*i*__^*l*^ and *c*__*i*__^*l*^ represent the selected atom at the *l*th node on path *S*_*i*_ and corresponding coefficient.

The search tree starts with less unspecified nodes. A simple way is selecting the *I* = *N* | 500 nodes that have the highest absolute inner product with Φ^*m*^. In order to find the fewest possible nodes, the search must constantly make an evaluation to decide which available paths should be expended next. Therefore, the evaluation function *g*(*S* ^*l*^) is defined as follows:

(6) $$\begin{array}{c} g\left(S^{l}\right)={||r^{l}||}_{2}={||\Phi^{m}-\sum_{j=1}^{l}c^{j}s^{j}||}_{2}. \end{array}$$

Beside the evaluation function, the auxiliary function is also needed to assess the cost brought by adding a preferred goal node to the path. Generally, according to the expectation on average equal contribution of unopened nodes, the auxiliary function can be built as follows:

(7) $$\begin{array}{c} d\left(S_{i}^{l}\right)\geq \left({||r_{i}^{l-1}||}_{2}-{||r_{i}^{l}||}_{2}\right)t, \end{array}$$

where coefficient *t* is defined as *t* = *αN* − *l*. *α* is a ratio between the number of the nonzero entries and the zero entries in the solution *X*. It is well known that the sparse solution has only less nonzero entries. Therefore, in most practical application, *α* is very smaller than 1. Here, we selected *α* = 0.005. If the source distribution could be seen as the *K*-sparse signal, *K*could be computed by *K* = ⌈*αN*⌉. The cost function can be written as follows:

(8) $$\begin{array}{c} f\left(S_{i}^{l}\right)=||r_{i}^{l}||-\beta \left(||r_{i}^{l-1}||-||r_{i}^{l}||\right)t, \end{array}$$

where *β* is a constant. A lot of experiments for different reconstruction models including 2D and 3D experiments were performed to evaluate the impact of *β* on the source reconstruction. We found that better results could be obtained when it varied in the interval [1.00, 1.25]. Therefore, in our experiments, its value was taken from the interval and set to be1.05.

In practice, if all of the children of the most promising partial path are added to the search tree at each iteration, the tree might have too many search paths. Therefore, the following pruning strategies are employed a guide on how the tree grows.

The first one is about extensions per path. At each step, it is not necessary to have all of the unopened atoms added to the current optimal path. We can expand the search tree only by the *B* children which have the highest absolute inner-product with the residue to the selected path. This pruning strategy decreases the upper bound to *B* ^*K*^ on the number of paths. Practically, *I* and *B* are selected to be much smaller than *N*, which can drastically decrease the paths involved in the search. Although the number of extensions per path is limited to *B*, it is also necessary to control the size of path. That is because that adding new paths at each iteration continues increasing required memory. Therefore, to reduce memory requirements, we adopted the “beam search” strategy [30] and limit the maximum number of paths in the tree by the beam width *P*. When this limit is exceeded, the paths with maximum cost are seen as the worst paths and are removed from the tree until *P* paths remain. Here, *B* and *P* were set to be 4 and 200, respectively. Moreover, since order of nodes along a path is unimportant, amalgamating the equivalent path is also important to improve the search performance. For this purpose, we define a path equivalency notion; *S*_1_^*l*_1_^ and *S*_2_^*l*_2_^ are two paths with different length *l*_1_ < *l*_2_. If all atoms of *S*_1_^*l*_1_^ can be found in *S*_2_^*l*_2_^ and these composed the continuous subset of *S*_2_^*l*_2_^, we define the above two paths as being equivalent. Consequently, the insertion of *S*_2_^*l*_2_^ into the tree is unnecessary.

After the growing of the search tree is finished, the linear relationship between the observation Φ^*m*^ and the selected PSR can be established as follows:

(9) $$\begin{array}{c} A_{\text{opt}}X=\Phi^{m}. \end{array}$$

Since the nodes contained in the optimal path can be much smaller than the number of all nodes *N*, (9) is an overdetermined linear equation. Therefore, a limited memory variation of the Broyden Fletcher Goldfarb Shanno (LBFGS) [31] was used to directly solve (9).

## 3. Simulation Studies in the Mouse Atlas

In this subsection, heterogeneous simulations were presented to demonstrate the performance of the proposed method for mouse applications. The experimental data were acquired by a dual-modality BLT/micro-CT system developed in our lab [32, 33]. By using image processing and interactive segmentation technology, heterogeneous model including heart, lungs, liver, bone, spleen, and muscle was built. The optical coefficients for each organ are listed in Table 1 [34]. Here, the torso section with a height of 25 mm was selected as the reconstruction region.

### 3.1. Reconstruction in a Single Source Case

**Figure 1:**
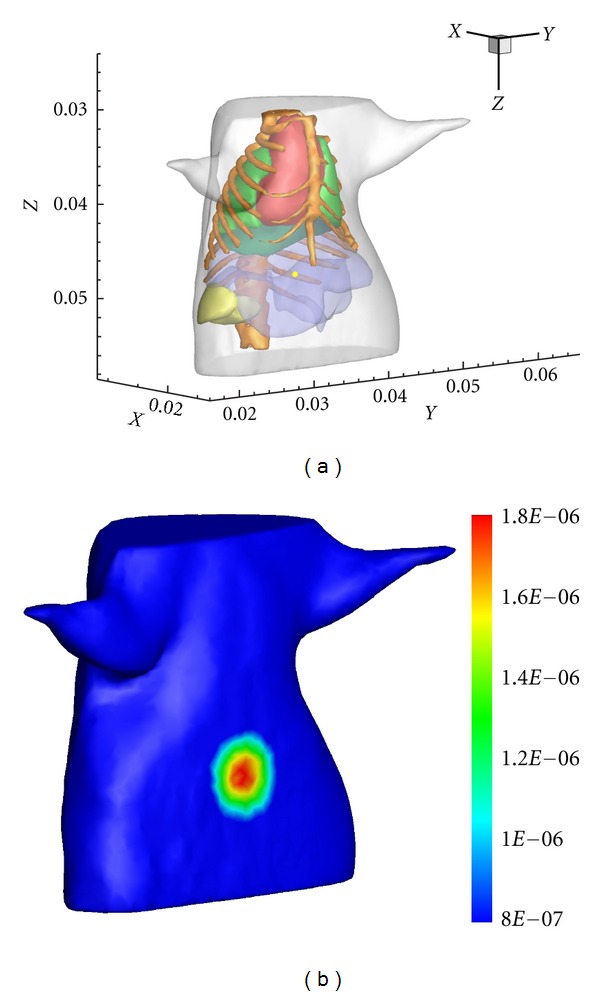
Reconstruction model with a single source. (a) The torso of the mouse atlas model with one source in the liver. (b) The simulated photon distribution on the surface.

In the experiment, a spherical source with a 0.6 mm diameter was placed in the liver with the center at (18.24 mm, 31.58 mm, 47.29 mm) as shown in Figure 1(a). The source was modeled as isotropic point sources whose strength was set to be 2 nW/mm^3^. As for the forward problem, the FEM was used to obtain the synthetic measurements on the boundary. The atlas model was discretized into a tetrahedral-element mesh with 30892 nodes and 167841 elements. The generated simulated photon distribution on the boundary is presented in Figure 1(b). Then the forward solutions were projected onto a single coarse mesh consisting of 20068 elements and 3098 nodes, which was used for reconstructing the source.

To better illustrate the performance of the proposed method, we compared the proposed method with OMP [25] and FIST-L1 [35, 36]. The former is a typical greedy pursuit method for sparse signal recovery. The latter, as a sparse regularization method, can be viewed as a standard approach to ill-posed linear inverse problems and has been used in fluorescence molecular tomography (FMT) and BLT. Here, the step size in FIST-L1 was computed by using the estimation algorithm introduced in [35]. Since the regularization parameter plays an important role in the regularization methods, we performed two experiments with different regularization parameters that were set to be 4e-11 and 4e-10, respectively. All of the reconstructions were carried out on a personal computer with 3.2 GHz Intel Core2 duo CPU and 2 GB RAM. 

**Figure 2:**
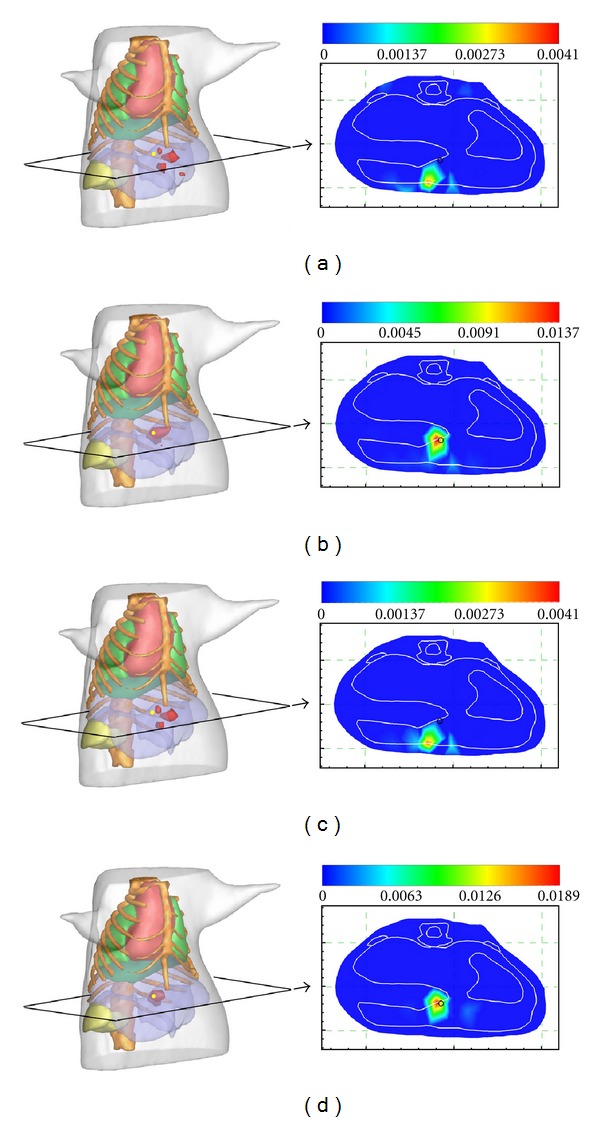
Comparison of reconstruction results. (a), (b), (c) and (d) are the reconstruction results with OMP, FIST-L1 (with regularization parameter set to be 4e-11), FIST-L1 (with regularization parameter set to be 4e-10), and the proposed method, respectively. The results are shown in the form of isosurfaces for 40% of the maximum value (left column) and slice images in *z* = 47.29 mm plane (right column). The small yellow sphere in the iso-surfaces view image and black circles in the slice images denote the real position of the bioluminescent source.

The qualities of the reconstruction were quantitatively assessed in terms of location error and the maximum reconstructed intensity. The location error was defined as Euclidean distance between *S*_real_ and *S*_recons_, where *S*_real_ and *S*_recons_ were the real locations of the source center and the location of the node with the maximum reconstructed value, respectively. The visual effects of the reconstruction results are presented in the form of slice images and iso-surfaces, as shown in Figure 2. Additionally, the detailed information about parameters and the final quantitative reconstruction results are summarized in Table 2.

We found that the reconstructed positions by L1 regularization with an optimal regularization parameter and the proposed method were identical. Specifically, the reconstructed center was (18.26 mm, 31.97 mm, 47.28 mm) with a location error of 0.3995 mm from the actual source, whereas the location error by OMP was 2.9605 mm. The performance of OMP was inferior to the other two methods. L1 regularization performed well and obtained satisfactory source localizations and maximum reconstruction value. However, the selection of the regularization parameter had a great impact on the reconstruction results. As for the proposed method, it performed slightly better than L1 regularization with manually optimized regularization parameter in terms of maximum reconstruction value. Moreover, it was also an efficient reconstruction method.

**Figure 3:**
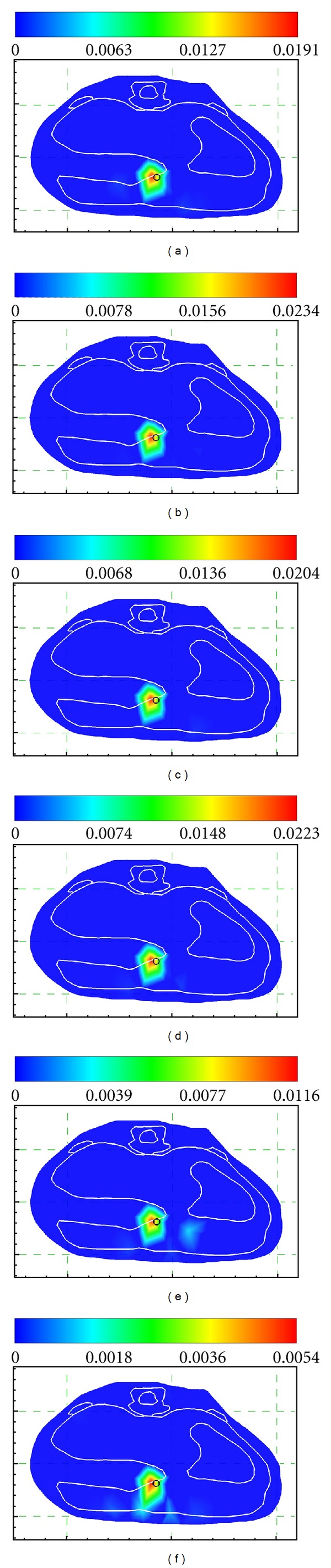
The proposed method for different noise levels of 0%, 5%, 10%, 15%, 20%, 25%, and 30%.

The above experiments were performed without noise. In order to evaluate the sensitivity of the proposed method to various noise levels, six cases were carried out where the measurements were added to 5%, 10%, 15%, 20%, 25% and 30% Gaussian noise, respectively. We also made a comparison between the proposed method and FIST-L1 with the regularization parameter that was set to be 4e-11. The reconstruction results under different noise levels are compiled in Table 3 and Figure 3 showing that the proposed method was robust to measurement noise.

### 3.2. Double Source Case and MultiSource Case

**Figure 4:**
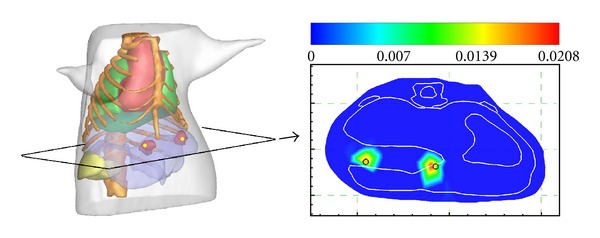
Reconstruction results in double source case. The results are shown in the form of iso-surface for 40% of the maximum value (left column) and slice image in *z* = 47.29 mm plane (right column). The small yellow sphere in the iso-surface view image and black circles in the slice image denote the real position of the bioluminescent source.

Dual source setting was also considered in order to evaluate the proposed method. Two sources have the same size as one used in the single source case. Their strength and position were set to be 2 nW/mm^3^ and (18.24 mm, 31.58 mm, 47.29 mm), 1 nW/mm^3^ and (18.74 mm, 39.15 mm, 47.06 mm), respectively. The reconstruction results are shown in Figure 4 and Table 4.

**Figure 5:**
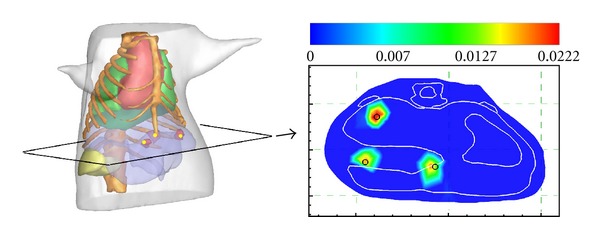
Reconstruction results in multisource case. The results are shown in the form of iso-surface for 40% of the maximum value (left column) and slice image in *z* = 47.29 mm plane (right column). The small yellow sphere in the iso-surface view image and black circles in the slice image denote the real position of the bioluminescent source.

Multiple sources setting simulation experiment was also presented to further demonstrate the ability of the proposed method. Based on the setting in double source case, the third source with the same size and shape was added. Its strength and position were set to be 1.5 nW/mm^3^ and (23.60 mm, 37.94 mm, 47.45 mm). The final reconstruction results are presented in Figure 5 and Table 5. The result of two group experiments indicated that the sources can be accurately distinguished by using the proposed method.

## 4. *In Vivo* Experiment Validation

Besides the numerical simulations with mouse atlas, an i*n vivo* experiment was carried out on a mouse to further test the proposed method. The experiment was also performed with a dual-modality BLT/micro-CT system developed in our lab [32, 33, 37]. A nude hairless mouse (Nu/Nu, Laboratory Animal Center, Peking University, China) was used in this experiment. To simulate a known bioluminescent source, a home-made cylindrical light source about 3 mm long and 1.5 mm in diameter was implanted into the abdomen of the mouse in this experiment. The source was made of a catheter filled with luminescent liquid and emitted a red luminescent light that had a similar emission spectrum with a firefly luciferase-based source.

**Figure 6:**
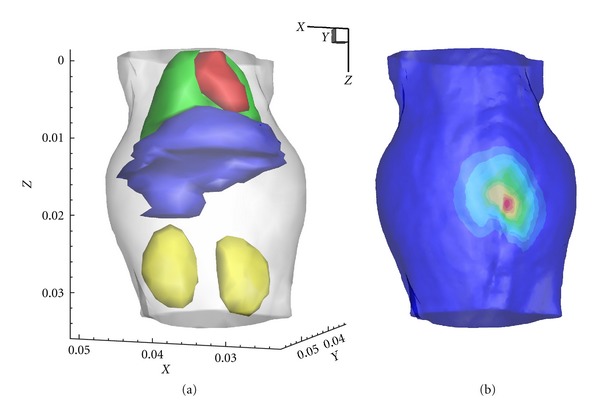
*In vivo* heterogeneous model. (a) The torso of the model. (b) The mapped photon distribution on the mouse surface.

Before the beginning of the experiment, the CCD (VersArray, Princeton Instruments, Trenton, NJ, USA) was cooled to 110° by using liquid nitrogen. The mouse was anesthetized and placed in a mouse holder. The mouse holder was set to rotate to 0°, 90°, 180° and 270°. At each of four positions, the mouse was photographed by the CCD camera. After the optical data were acquired, the mouse was scanned by using the micro-CT to obtain the anatomical maps which could provide structural information for the source reconstruction. Then the CT data were segmented into five regions represent muscle, lungs, heart, liver, and kidneys, respectively, as shown in Figure 6(a). The heterogeneous model including five tissues was discretized into the mesh containing 11917 tetrahedral elements and 2557 nodes. The optical parameters for different tissues were calculated based on the literature as listed in Table 6 [34, 38]. The optical data was registered with the volumetric mesh, and measured data were mapped onto the surface of the mesh. The result of mapped photon distribution is shown in Figure 6(b).

**Figure 7:**
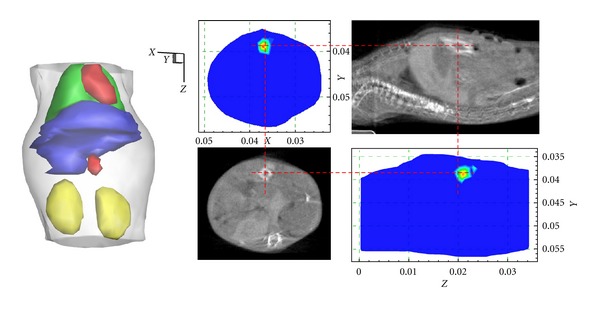
The reconstruction results. The results are shown in the form of isosurface for 40% of maximum value (left column) and slice images (right column). The transverse view of the results and the comparisons with the corresponding CT slices. The cross of the red lines denotes the actual source center.

It took about 8 seconds to complete the reconstruction using the proposed method. The final results are presented in Figure 7, where the reconstruction source center is (37.17 mm, 38.82 mm, and 20.92 mm) with a deviation about 2 mm to the actual center. As can be seen in the reconstruction results, the proposed methods could obtain satisfactory bioluminescent source localizations.

## 5. Discussion and Conclusion

In this paper, we have proposed a new method based on the semigreedy for bioluminescence tomography. The reconstruction results of the simulations on a mouse atlas demonstrate that the proposed reconstruction method is able to accurately and stably localize bioluminescent source from whole body, even with noisy measurements. The *in vivo* experiment further shows its performance.

The PSR strategy can significantly improve reconstruction qualities. However, in most cases, empirically selecting such small and appropriate region is unconvenient, even available. In this study, The optimal PSR problem is cast into a search for the correct support of source distribution among a number of dynamically evolving candidate subsets. In view of the characteristics in BLT sparse distribution, only the columns that contribute to the source reconstruction are chosen automatically by using semi-greedy method. The constraint imposed on the source space reduces the ill posedness of the problem and computational cost.

 It is noted that *in vivo* experiment is not as accurate as simulations. Some reasons can be explained for this phenomenon. First of all, the error was generated, when the energy distribution was mapped from 2D images to a 3D mouse surface. Secondly, there were only five main segmented tissues used to build a heterogeneous model while others simply were regarded as the muscle, which also led to errors. Finally, the accuracy of the photon propagation model was very important for source reconstruction. The diffusion approximation was used due to its moderate computational efficiency and explicit physical meaning. However, it has some limitations in certain regions, such as void or more absorptive regions. Therefore, the error brought on by the DA model is inevitable. As discussed above, our future work will focus on studying more accurate forward models to describe photon propagation in biological tissues and improving the experimental procedures and imaging system to further promote the reconstruction quality.

## Figures and Tables

**Table 1:** Optical parameters for each organ in the mouse atlas [34].

	Muscle	Heart	Lungs	Liver	Spleen	Bone
*μ*_*a*_ (mm^-1^)	0.032	0.022	0.071	0.128	0.075	0.002
*μ*_*s*_′ (mm^-1^)	0.586	1.129	2.305	0.646	2.178	0.935

**Table 2:** Quantitative comparisons of the reconstruction results.

Method	Recon. location center (mm)	Location error (mm)	Recon. time (s)	Maximum recon. value (nW/mm^3^)
OMP	(15.61, 32.92, 47.48)	2.9605	0.798	0.00412
FIST-L1(1)	(18.26, 31.97, 47.28)	0.3995	16.94	0.01371
FIST-L1(2)	(15.61, 32.92, 47.48)	2.9605	25.44	0.00414
The proposed method	(18.26, 31.97, 47.28)	0.3995	12.20	0.01889

**Table 3:** Quantitative results in a single source case with different noise levels.

Method	Noise level	Recon. location center (mm)	Location error (mm)	Recon. Time (s)	Maximum recon. value (nW/mm^3^)
FIST-L1	5%	(18.26, 31.97, 47.28)	0.3995	24.43	0.0122
	10%	(18.26, 31.97, 47.28)	0.3995	24.99	0.0085
	15%	(18.26, 31.97, 47.28)	0.3995	24.77	0.0050
	20%	(15.61, 32.92, 47.48)	2.9605	24.82	0.0032
	25%	(15.61, 32.92, 47.48)	2.9605	24.94	0.0025
	30%	(15.61, 32.92, 47.48)	2.9605	24.60	0.0023

The proposed method	5%	(18.26, 31.97, 47.28)	0.3995	13.41	0.0192
	10%	(18.26, 31.97, 47.28)	0.3995	14.15	0.0234
	15%	(18.26, 31.97, 47.28)	0.3995	16.37	0.0204
	20%	(18.26, 31.97, 47.28)	0.3995	18.09	0.0223
	25%	(18.26, 31.97, 47.28)	0.3995	18.95	0.0116
	30%	(18.26, 31.97, 47.28)	0.3995	46.61	0.0054

**Table 4:** Reconstruction results in double source case.

Source number	Actual position (mm)	Recon. location center (mm)	Location error (mm)	Maximum recon. value (nW/mm^3^)
1	(18.24, 31.58, 47.29)	(18.26, 31.97, 47.28)	0.3995	0.0204
2	(18.74, 39.15, 47.06)	(18.75, 39.20, 46.86)	0.2064	0.0208

**Table 5:** Reconstruction results in multiple source case.

Source number	Actual position (mm)	Recon. location center (mm)	Location error (mm)	Maximum recon. value (nW/mm^3^)
1	(18.24, 31.58, 47.29)	(18.26, 31.97, 47.28)	0.3995	0.0195
2	(18.74, 39.15, 47.06)	(18.75, 39.20, 46.86)	0.2064	0.0226
3	(23.60, 37.94, 47.45)	(23.60, 37.97, 47.65)	0.2023	0.0300

**Table 6:** Optical parameters for each organ in the heterogeneous model [34, 38].

	Muscle	Heart	Lungs	Liver	Kidneys
*μ*_*a*_ (mm^-1^)	0.008	0.138	0.456	0.829	0.150
*μ*_*s*_′ (mm^-1^)	1.258	1.076	2.265	0.735	2.507
